# Validation of methods for converting the original Disease Activity Score (DAS) to the DAS28

**DOI:** 10.1007/s00296-018-4184-0

**Published:** 2018-10-27

**Authors:** Lewis Carpenter, Sam Norton, Elena Nikiphorou, Patrick Kiely, David A. Walsh, Josh Dixey, Adam Young

**Affiliations:** 1 Health Psychology Section, King’s College London, London, UK; 2 Department of Rheumatology, King’s College London, London, UK; 3 Department of Rheumatology, St George’s University Hospital NHS Foundation Trust, London, UK; 4 Arthritis UK Pain Centre, University of Nottingham, Nottingham, UK; 5 Department of Rheumatology, Wolverhampton NHS Trust, Wolverhampton, UK; 6 Postgraduate Medicine, University of Hertfordshire, Hatfield, UK

**Keywords:** Rheumatoid arthritis, Epidemiology, Observational studies, Statistical methods

## Abstract

The Disease Activity Score (DAS) is integral in tailoring the clinical management of rheumatoid arthritis (RA) patients and is an important measure in clinical research. Different versions have been developed over the years to improve reliability and ease of use. Combining the original DAS and the newer DAS28 data in both contemporary and historical studies is important for both primary and secondary data analyses. As such, a methodologically robust means of converting the old DAS to the new DAS28 measure would be invaluable. Using data from The Early RA Study (ERAS), a sub-sample of patients with both DAS and DAS28 data were used to develop new regression imputation formulas using the total DAS score (univariate), and using the separate components of the DAS score (multivariate). DAS were transformed to DAS28 using an existing formula quoted in the literature, and the newly developed formulas. Bland and Altman plots were used to compare the transformed DAS with the recorded DAS28 to ascertain levels of agreement. The current transformation formula tended to overestimate the true DAS28 score, particularly at the higher end of the scale. A formula which uses all separate components of the DAS was found to estimate the scores with a higher level of precision. A new formula is proposed that can be used by other early RA cohorts to convert the original DAS to DAS28.

**Electronic supplementary material:**

The online version of this article (10.1007/s00296-018-4184-0) contains supplementary material, which is available to authorized users.

## Introduction

The Disease Activity Score (DAS) is a composite score developed as a means of quantifying the severity of rheumatoid arthritis (RA) [1]. It is central in the current ‘treat-to-target’ (T2T) paradigm [2]. It is the most widely used ‘tool’ in clinical practice to assess and monitor the patients’ disease activity status and to help guide treatment adjustments. In the UK, the DAS28 is one of the main determinants in deciding access to biologic disease-modifying antirheumatic drugs (bDMARDs) [3].

Several versions of the DAS exist. The original DAS established that four measures could be combined to give an effective overview of the overall disease status of a patient: a 44 joint Swollen Joint Count (SJC), the Ritchie Articular Index (RAI) of 53 tender joints, the Erythrocyte Sedimentation Rate (ESR) and a Patient Global Assessment (PGA) of disease activity on a 100 mm visual analogue scale (VAS) [1]. A three-variable calculation excluding the PGA is also available. In 1995, a modified version of the DAS was proposed, the DAS28, which used counts of 28 joints for both the SJC and tender joint count (TJC) focusing on the hands and arms [4]. Discrepancies between the two DAS scoring methods has been shown to occur, in part, by the omission of the feet and ankles in the DAS28 joint count [5]. However, the DAS28 is able to discriminate between patients with varying levels of disease activity and was preferred due to its simplicity and feasibility in the clinical setting. Other variations of the DAS and DAS28 exist, including the use of C-reactive protein (CRP) in place of ESR. However, the original four-variable DAS and DAS28 using ESR remain the most routinely used methods [6], and are part of the core set of outcomes in RA clinical trials [7].

It is agreed that the DAS and DAS28 scores cannot be used inter-changeably due to their differences in minimum and maximum values, and weighting used for each component of the score [6]. However, comparing these data would provide the opportunity to study longitudinal cohorts where different DAS methods have been used (e.g., Norfolk Arthritis Register (NOAR) [8] and the Yorkshire Early Arthritis Register (YEAR) [9], along with many other registries worldwide [10]), and to enable meta-analyses across studies reporting different DAS outcomes. This was the case in early data collected from The Early RA Study (ERAS), which collected the three-variable DAS for patients diagnosed between 1986 and 2001, while the Early RA Network (ERAN) collected DAS28 data for patients diagnosed between 2002 and 2011 [11].

Methods for transforming the original DAS to the DAS28 metric have been proposed, and used in a number of previous studies to try and facilitate direct comparisons [11–14]. A commonly used formula was devised by van Gestel et al. in 1998 using longitudinal data from an observational cohort [15]. Although precise details about how the formula was derived are not shown, it is likely to represent a regression imputation of the original DAS regressed on DAS28 scores to impute the missing DAS28 scores. While DAS and DAS28 scores are known to correlate highly, there is currently no research to assess the reliability and validity of this imputation method.

Using a sub-group of patients from the ERAS cohort attending one centre where both DAS and DAS28 data are available, this paper explores the use of imputation methods, including the formula given by van Gestel et al., to calculate missing DAS28 scores where DAS are recorded. The analyses will compare levels of agreement of (1) the van Gestel formula, (2) univariable regression imputation using DAS to predict DAS28, and (3) multivariable regression imputation model using the separate components of DAS to predict DAS28. Finally, the paper will examine differences in trends over time using the different methods to calculate DAS28 to determine potential differences in interpretation. The aim is to provide a potentially more statistically reliable transformation formula for converting DAS to DAS28.

## Methods

### Data

The Early RA Study (ERAS) recruited a total of 1465 patients with early RA from nine centres in England between 1986 and 2001. All patients had a confirmed diagnosis of RA and were recruited within 2 years of symptom onset (median 6-months), prior to conventional DMARD initiation. All patients were treated based on standard clinical practice of the time. Standard clinical, laboratory and radiographic data were collected during outpatient appointments at baseline, 6 months, 12 months, and then yearly thereafter. Each centre closed to follow up at different times between 2001 and 2013 with a maximum follow-up of 30 years (median 10 years) for annual outpatient follow-up. ERAS received retrospective ethical approval from the East Hertfordshire Local Research Ethics Committee in 1994 and subsequently from the Caldicott Guardian.

The analysis uses a subset of the main ERAS dataset with data from one centre (*n* = 298), where the component variables for both the DAS and DAS28 were recorded for the same patients by the same examiner. Restricting the analyses to the first 5 years, the 298 patients contributed a total of 1470 observations (mean 4.9 observations per patient).

### Disease activity scores

All patients in ERAS had the 44-count SJC, the RAI, and ESR collected at each visit. The PGA was not routinely collected by centres, so the three-variable DAS formula was used to calculate the original DAS for all patients [1] (Eq. 1).

1 $${\text{DAS}}(3)=\left( {0.54 \times {\text{sqrt}}({\text{RAI}})} \right)+0.065 \times ({\text{SJC}}44)+0.33 \times \ln ({\text{ESR}})+0.22.$$

For the subset of patients where the DAS28 component variables were collected, DAS28 was calculated using the four-variable formula [4] (Eq. 2)

2 $${\text{DAS}}28(3){\text{ }}={\text{ }}\left( {0.56 \times {\text{sqrt}}({\text{TJC}}28)} \right){\text{ }}+{\text{ }}\left( {0.28 \times {\text{sqrt}}({\text{SJC}}28)} \right){\text{ }}+{\text{ }}0.70\ln ({\text{ESR}}) \times 1.07{\text{ }}+{\text{ }}0.14 \times {\text{PGA}}.$$

While a PGA VAS was not routinely collected, patient assessment of global pain using a 0–100 VAS was. PGA was used where available but where missing (94% observations) the pain VAS was used in place. The large proportion of missing PGA data was due to the fact that PGA data was typically collected beyond the 5-year follow-up for patients recruited from this centre. Khan et al. indicates that different measures of global health can be used inter-changeably without significant impact on the DAS [16]. This is likely due to the very high correlation between PGA and other measures of patient global health (i.e., PGA with pain VAS correlation was *r* = .900 in the current data). Sub-analysis (not shown) found that there was higher agreement between the DAS28 and DAS28 using pain VAS compared to just the three-variable DAS28 score, therefore the four-variable DAS28 with either the PGA or pain VAS was used.

The conversion formula given by van Gestel et al. [15] for imputing DAS28 based on DAS is shown in Eq. (3). The intention of the formula is to convert the DAS score to the DAS28 metric, however, it should be noted that essentially, as the multiplication factor is close to one, the formula simply increases the DAS score by 0.9 units.

3 $${\text{DAS}}28~=~((1.072) \times {\text{DAS}})~+~0.938.$$

### Analysis

The analysis examines three methods of imputing DAS28. Whilst other imputation methods have proven more effective in imputing missing data, such as multiple imputation [17], this is typically not possible in the context of transforming DAS to DAS28, as no data is available for the DAS28. As such, this paper focuses only on the use of regression imputation methods to devise a formula for wider use.

Univariable mixed-effects linear regression models regressed the DAS28 on to the DAS for the DAS-only regression imputation. Mixed models were used to account for the longitudinal nature of the data. The fixed effects from the model, the coefficient for DAS and the constant, were then used to calculate imputed DAS28 scores. A similar procedure was applied for the multivariable mixed-effects linear regression imputation using the individual DAS components: the square-root of the 44-count SJC and the RAI, the natural logarithm of ESR and PGA/pain VAS scores will be used. Additionally, sex (coded male 1 and female 2) and age in years were also included as predictors, since these are known to independently impact on ESR [18]. The coefficient of each predictor, along with the constant, was then used to calculate imputed DAS28 scores. To evaluate the performance of the models, the model fit statistics were examined.

Mean scores of the DAS28 over the first 5 years will be compared to the three imputation methods outlined, and the mean difference, along with levels of agreement, will be assessed using Bland and Altman plots [19]. Bland and Altman plots are an effective way of assessing agreement between two scores and plots the difference between the two scores against the average of both scores. The Bland and Altman plots also provide summary statistics on the agreement between the scores using the mean difference, as well as providing 95% limits of agreement (LoA) around this mean estimate. Much like a 95% confidence interval, these limits are approximately 2 standard deviations above and below the mean difference. The closer the mean difference is to zero, the higher the agreement. The narrower the 95% LoA, the less variation there is around this mean estimate [19]. The intra-class correlation coefficient (ICC) will also be used as another measure of agreement between each imputation method and the actual DAS28 scores. Finally, the DAS28 will be categorised into remission, low disease activity (LDA), moderate disease activity (MDA) and high disease activity (HDA) according to the EULAR DAS28 thresholds [20]. The proportion of patients categorised into each group will be assessed graphically, and agreement across categories will be assessed using the Kappa statistic [21].

## Results

### Demographic data of patient sub-sample

Of the patients from ERAS (*n* = 1465), 298 (20.3%) attending one centre had the component variables for both DAS and DAS28 collected. The 298 patients contributed 1470 observations over 5 years (mean 4.9 observations per patient). Demographic and baseline clinical measures for the whole cohort, and those with and without DAS28 measures over the first 5 years are shown in Table 1. Patients with both DAS and DAS28 data were slightly older, more likely to be female and less likely to be seropositive. Baseline HAQ levels were similar across the groups, however, patients with recorded DAS28 indicated higher levels of baseline DAS scores when compared to the rest of the cohort. This is reflected by increased SJC, RAI and pain VAS scores at baseline, although baseline ESR was marginally lower.

**Table 1:** Descriptive Statistics of ERAS cohort with and without DAS data

	Total		ERAS (DAS only)		ERAS (DAS and DAS28)	
	*N* = 1465		*N* = 1167		*N* = 298	
Age at onset (years)						
Mean (standard deviation)	55.3 (14.57)		54.62 (14.36)		57.97 (15.09)	
Female, *n* (%)	973 (66.42%)		769 (65.90%)		204 (68.46%)	
Baseline DAS						
Mean (standard deviation)	4.23 (1.63)		4.07 (1.51)		4.85 (1.91)	
Median (inter-quartile range)	4 (2)		4 (2)		5 (3)	
Baseline SJC-44						
Mean (standard deviation)	17.54 (13.01)		16.1 (11.80)		23.19 (15.72)	
Median (inter-quartile range)	14 (19)		13 (18)		22 (25)	
Baseline RAI						
Mean (standard deviation)	12.63 (11.11)		11.64 (10.45)		16.48 (12.69)	
Median (inter-quartile range)	10 (12)		9 (11)		14 (18)	
Baseline pain VAS						
Mean (standard deviation)	43.97 (26.37)		43.61 (26.11)		45.31 (27.30)	
Median (inter-quartile range)	45 (40)		45 (38)		45 (43)	
Baseline ESR						
Mean (standard deviation)	42.17 (28.79)		42.53 (28.88)		40.79 (28.42)	
Median (inter-quartile range)	37(44)		38 (44)		34 (39)	
Baseline HAQ						
Mean (standard deviation)	1.15 (0.77)		1.16 (0.77)		1.1 (0.77)	
Median (inter-quartile range)	1 (2)		1 (2)		1 (2)	
Seropositivity, *n* (%)	914 (62.39%)		744 (63.75%)		170 (57.05%)	

*DAS* Disease Activity Score, *SJC* swollen joint count, *RAI* Ritchie Articular Index, *VAS* Visual Analogue Scale, *ESR* erythrocyte sedimentation rate, *HAQ* Health Assessment Questionnaire

### Regression imputation

The fixed-effect estimates from the mixed-effects linear regressions for the univariable and multivariable regression imputations are shown in Table 2. The effect estimates give the following imputation formula for the univariate regression of DAS28 on DAS:

**Table 2:** Regression output from univariable and multivariable regression models

	Univariable imputation	Multivariable imputation
Observations	1470	1470
*N*	298	298
DAS (95% CI)	0.736 (0.71, 0.76)	
SJC44 (95% CI)		0.3 (0.27, 0.33)
RAI (95% CI)		0.175 (0.14, 0.21)
ESR (95% CI)		0.739 (0.71, 0.77)
PGA (95% CI)		0.016 (0.01, 0.02)
Sex (95% CI)		− 0.027 (− 0.11, 0.06)
Age (95% CI)		− 0.001 (− 0.00, 0.00)
Constant (95% CI)	1.247 (1.13, 1.37)	− 0.086 (− 0.31, 0.14)
Psuedo *R*^2^	0.73	0.91

*DAS* Disease Activity Score, *SJC* swollen joint count, *RAI* Ritchie Articular Index, *ESR* erythrocyte sedimentation rate, *PGA* patient global assessment

$${\text{DAS}}28~=~({\text{DAS}} \times 0.736)~+~1.247.$$

The effect estimates from the multivariable regression of DAS28 on the square-root 44-SJC and RAI, the log ESR, pain VAS, age and sex are as follows:

4 $${\text{DAS}}28~=~~({\text{sqrt}}({\text{SJC}}44) \times 0.300)~+~({\text{sqrt}}({\text{RAI}}) \times 0.175)~+~({\text{log}}({\text{ESR}}) \times 0.739)~+~({\text{PGA}} \times 0.016)~+~({\text{female}} \times - 0.027)~+~({\text{age}} \times - 0.001)~+~ - 0.086.$$

The estimated pseudo *R*^2^ for the univariable regression and multivariable regression model was 0.73 and 0.91, respectively, indicating high predictive accuracy. This was further supported by the results of the cross-fold validations (shown in supplementary material 1) which indicate similar pseudo *R*^2^ across the models for the univariable and multivariable regression: 0.72 and 0.91, respectively.

**Fig. 1:**
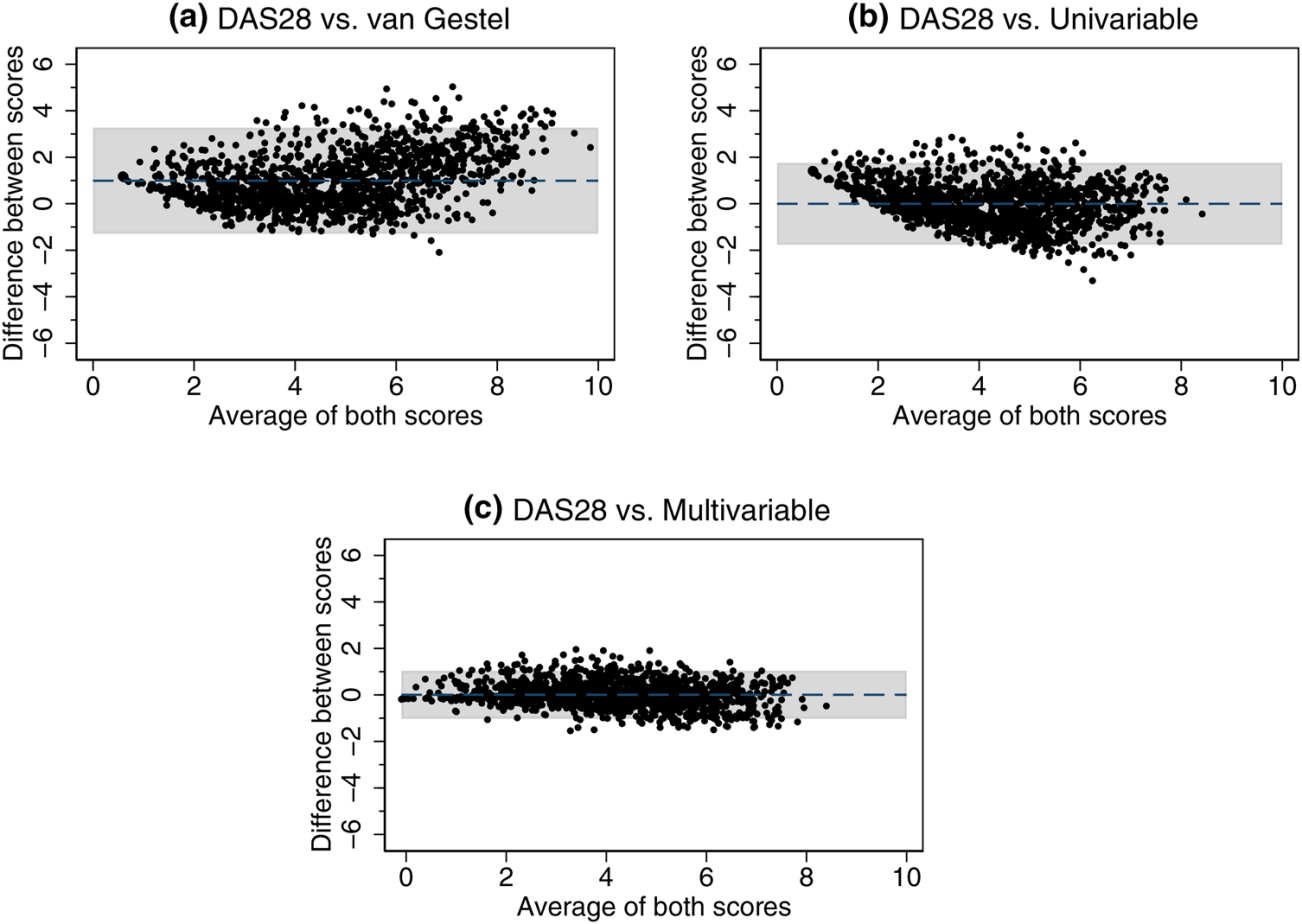
**a**–**c** Bland and Altman plots testing the agreement between **a** DAS28 score and the van Gestel transformation, **b** DAS28 score and the univariable method, and **c** DAS28 score and the multivariable method

**Fig. 2:**
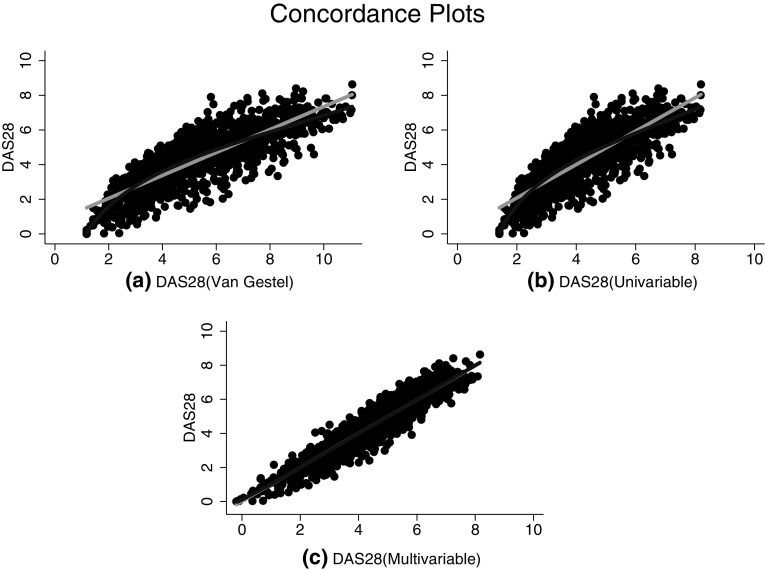
**a**–**c** Scatter concordance plots between **a** DAS28 score and the van Gestel transformation, **b** DAS28 score and the univariable method, and **c** DAS28 score and the multivariable method. The light grey line indicates the linear fit, and the dark grey line indicated the best fitting non-linear line (fractional polynomial)

Bland and Altman plots (Fig. 1a–c) were used to investigate the level of agreement between the recorded DAS-28 scores and each of the three imputation methods. The largest bias was found for the van Gestel formula at 0.99, along with the widest limits of agreement (LoA) at − 1.29 to 3.27. The univariable regression imputation exhibited no bias (mean difference of − 0.00), with slightly narrower LoA at − 1.76–1.76, whilst the multivariable regression imputation also exhibited no bias (mean difference of 0.01), along with the narrowest LoA at − 1.02 to 1.04. The plots highlight how for both the van Gestel and univariable regression method, there is a tendency for the lower scores to be overestimated. In fact, for both methods the minimum imputed DAS28 score observed is approximately one. Scatter plots showing concordance between each imputation method and the DAS28 score are shown in Fig. 2a–c. The red line is the reduced major axis of the data, which is equivalent to the slope of the correlation between the variables. The blue line shows the best fitting (non-linear) fractional polynomial line. This also highlights how both the van Gestel and univariable method overestimate true DAS28 scores at the lower and higher end of the scale, whereas the multivariable method indicates much closer agreement across the whole range of the score.

The Pearson correlation between the DAS28 and van Gestel formula was 0.85, for the univariable regression was 0.85 and for the multivariable regression was 0.95. The ICC for the transformation formula was the lowest at 0.72 (95% confidence interval 0.69–0.74), followed by the regression imputation using only the DAS at 0.84 (95% CI 0.83–0.86), and finally the highest ICC was observed for the multiple regression imputation with the component scores at 0.95 (95% CI 0.95–0.96).

When categorised into remission, LDA, MDA and HDA, the recorded DAS28 scores indicated 22% of scores in remission, 12% of scores in LDA, 38% of scores in MDA and 28% of scores in HDA. The van Gestel formula saw an increased proportion of scores recorded as HDA at 45%, with lower proportions in the other groups. Conversely, both the univariable imputation and the multivariable imputation indicated slight underestimation in the remission and HDA categories, and slight overestimation in the LDA and MDA categories.

The weighted Kappa agreement analysis of the DAS28 categories indicated the highest level of agreement between the multivariable imputation at 97.6% and a Kappa statistic of 0.91. Univariable imputation using only the DAS score indicated an agreement of 95.2%, with a Kappa statistic of 0.81, whilst the transformation formula method indicated the lowest agreement at 93.6% and a Kappa statistic of 0.77.

### DAS imputations over time

**Fig. 3:**
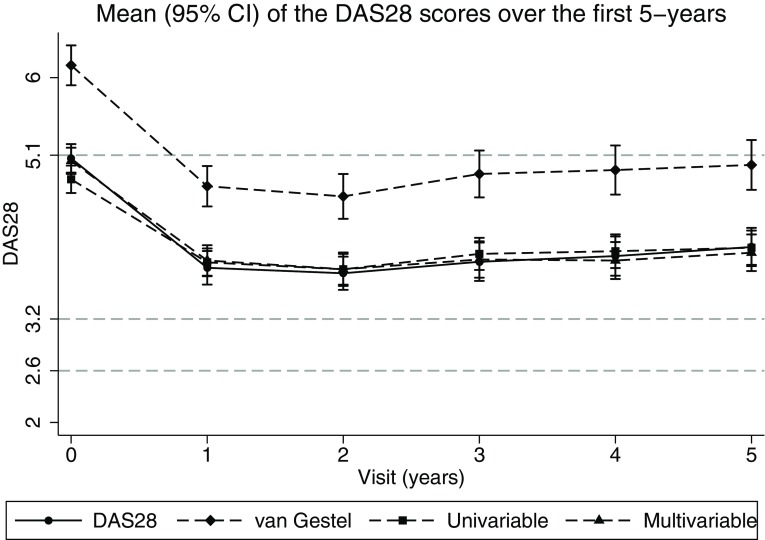
Mean and 95% Confidence Intervals over the first 5-years of the van Gestel transformation, univariable method and multivariable method, along with the recorded Disease Activity Score (DAS) 28

The overall mean of the DAS28 over the first 5 years was 4.10 (SD 1.7). This compared to an overall mean of 5.09 (SD 2.19) using the van Gestel formula, 4.10 (SD 1.51) using univariable regression imputation of DAS and 4.11 (SD 1.63) using the multivariable regression imputation with the separate DAS components. While the mean scores were similar for the univariable and multivariable regression scores, the variability in the DAS28 was better approximated by the multivariable imputation model. The mean and standard deviations for each method over 5 years follow-up is given in Table 3. Figure 3 indicates the mean scores, along with the 95% confidence interval of the DAS28, the van Gestel formula, the univariable imputation and the multivariable imputation.

**Table 3:** Mean (SD) of the recorded DAS28 score, along with the transformed DAS28 score using the van Gestel formula, univariable imputation and multivariable imputation

	Total		Baseline		1 year		2 years		3 years		4 years		5 years	
	*N* =1499		*N* = 295		*N* = 237		*N* = 257		*N* =243		*N* =237		*N* =230	
DAS28														
Mean (standard deviation)	4.1 (1.70)		5.06 (1.45)		3.79 (1.53)		3.73 (1.57)		3.86 (1.77)		3.93 (1.79)		4.04 (1.72)	
DAS28 van Gestel formula														
Mean (standard deviation)	5.09 (2.19)		6.14 (2.05)		4.69 (1.96)		4.63 (2.11)		4.89 (2.21)		4.94 (2.23)		5.01 (2.22)	
DAS28 univariable imputation														
Mean (standard deviation)	4.1 (1.51)		4.82 (1.41)		3.82 (1.35)		3.78 (1.45)		3.96 (1.52)		3.99 (1.53)		4.04 (1.53)	
DAS28 multivariable imputation														
Mean (standard deviation)	4.11 (1.63)		5.03 (1.34)		3.84 (1.49)		3.78 (1.58)		3.89 (1.71)		3.89 (1.67)		3.98 (1.64)	

*DAS* Disease Activity Score

## Discussion

Using a sub-sample of early RA patients from a longitudinal cohort, this study found that multivariable regression imputation of the DAS28 using the separate components of the original DAS measure resulted in the highest level of agreement when compared with recorded DAS28 scores. Furthermore, using the formula provided by van Gestel et al. [15], resulted in the lowest levels of agreement in this dataset. Whilst univariable regression imputation using only the total DAS score provided an improvement over the formula provided by van Gestel et al. [15], it showed similar levels of variability in the agreement when compared to the van Gestel transformation formula, across the spectrum of low to high scores.

The relatively large improvement in the mean difference between the univariable regression imputation using the total DAS in this analysis, compared to the formula by van Gestel et al. [15] suggests that using regression imputation formulae from different datasets may lead to some bias in the calculation of the transformed scores. Over the course of the first 5 years, the DAS28 using this formula was consistently more than one unit greater than the true DAS28 score, which resulted in much higher proportion of patients categorised into the HDA group. It is unclear why the univariable regression imputation devised using the ERAS data differed quite so significantly from the formula by van Gestel et al. [15], but it may be in part due to fundamental differences between the patient populations, such as differences between Dutch and UK patients, disease duration or disease severity that have impacted on the correlation between the two variables. Thus, ERAS is a cohort of patients with newly diagnosed RA where the components of the DAS will be less influenced by damage as would become apparent in patients with more longstanding disease. Unfortunately, the details of the derivation of the van Gestel formula are not clear, making it impossible to interrogate fully.

The analyses demonstrated that performing multivariable regression imputation using the separate components of DAS within the same dataset is the best approach for transforming DAS to DAS28. This is useful in situations where the DAS has been recorded but the DAS28 is preferred. Such instances include longitudinal studies considering secular trends over time [11, 14] and meta-analyses [22, 23]. In such instances, where the DAS components are known, the authors recommend the use of the multivariable regression imputation formula outlined in this analysis in datasets of early RA, as this demonstrated greater agreement when compared to the van Gestel formula or the use of the univariable regression imputation formula.

Other forms of imputation, such as multiple imputation using chained equations (MICE) are also available, that have been shown to be less biased and more robust in imputing missing data [17]. Sensitivity analysis using a MICE approach was conducted (results not shown), however, it was not found to have worse levels of agreement compared to the multivariable regression imputation method outlined. This is to be expected since MICE treats the DAS28 score as unobserved, which introduces Monte Carlo error into the estimates. Given the additional steps involved in the calculation and use of MICE and multiply imputed data, the authors recommend that it should only be used in cases where individual components of the DAS28 are missing, to allow for more complete calculation of the DAS28 measure. In such cases, it is important that a measure of uncertainty around the imputation estimates is also given. However, for instances where only the original DAS is measured, the multivariable regression imputation method and formula outlined can be used.

Examination of demographic and baseline clinical variables for the sub-sample of patients with both DAS and DAS28 data indicated that, on average, these patients had higher disease activity at presentation. This could therefore indicate that the imputations were based on a sub-group of patients with more severe forms of RA. Interestingly, incidence of seropositivity and levels of ESR were lower than the rest of the cohort, suggesting that higher DAS may have been influenced more by TJC and pain VAS, the so-called subjective markers, rather than the more objective markers of inflammation [23, 24]. Nevertheless, it does suggest that these patients from which the multivariable regression imputation was derived, represent a marginally different sub-type of RA at presentation. Furthermore, the use of the pain VAS rather than the PGA VAS score for the majority of the DAS28 scores may have influenced the results. However, previous research has demonstrated that different measures of global health can be used interchangeably without significant impact on the DAS measure [16]. This, along with the very high agreement between the pain VAS and PGA VAS suggests that any bias is likely to be low from adopting this method. Moreover, this study represents the first analysis to investigate the use of different DAS scores in RA using one of the largest datasets of early RA patients, with long-term follow-up.

In summary, the analysis presented demonstrates the advantage of using multivariable regression imputation in situations where older data using DAS are to be converted to DAS28, to facilitate aggregation of data. While the best method is to use estimates from a regression model derived from the data itself, the paper provides a formula to transform data based on the separate components of the DAS, rather than just the overall DAS itself, in instances where data-specific parameters are not available. This formula provides an improved and statistically more reliable method for converting DAS to DAS28 for long-term analysis where research necessitates the combination of historical and contemporary data in RA.

## Electronic supplementary material

Below is the link to the electronic supplementary material.

Supplementary material 1 (DOCX 57 KB)
