# Supplementary material for: Validation of methods for converting the original Disease Activity Score (DAS) to the DAS28
Source: Rheumatol Int. 2018 Oct 27;38(12):2297–305. doi: 10.1007/s00296-018-4184-0 (PMC6223857; doi:10.1007/s00296-018-4184-0)
Supplement: Supplementary file 1 — Supplementary material 1 (DOCX 57 KB) [file 296_2018_4184_MOESM1_ESM.docx]

**Supplementary Material 1**

Results of the univariable mixed-effects linear regression models regressing DAS on DAS28, using 5 cross-fold validation on 20% of the data:

Results of the multivariable mixed-effects linear regression models regressing square-root SJC-44, square-root TJC RAI, natural log ESR, PGA, Sex (Female) and age on DAS28, using 5 cross-fold validation on 20% of the data:
